# Circulating serum 25-hydroxyvitamin D levels and bone mineral density and osteoporotic vertebral fractures: A Mendelian randomization study

**DOI:** 10.1515/jtim-2026-0058

**Published:** 2026-06-22

**Authors:** Lihui Chen, Jiao Zhao, Zhenlin Zhang, Chao Gao

**Affiliations:** Department of Osteoporosis and Bone Disease, Shanghai Clinical Research Center of Bone Disease, Sixth People's Hospital, Shanghai Jiao Tong University School of Medicine, Shanghai, China

**Keywords:** 25-hydroxyvitamin D, bone mineral density, vertebral fractures, mendelian randomization, single nucleotide polymorphisms

## Abstract

**Background and Objectives:**

Low serum 25-hydroxyvitamin D (25OHD) has been associated with osteoporosis and osteoporotic fracture, but whether these associations are causal remains unclear.

**Methods:**

We identified 15 single-nucleotide polymorphisms (SNPs) affecting serum 25OHD levels in seven genes in the peripheral blood genomic deoxyribonucleic acid (DNA) of 2061 community-based people aged 20–92 years (711 men and 1350 women) and 200 outpatients aged 55–85 years with vertebral fractures (36 men and 164 women). Our study employs Mendelian randomization (MR) to determine the causal relationship between serum 25OHD concentration and bone mineral density (BMD), bone turnover markers (BTMs), and vertebral fracture.

**Results:**

MR analysis indicated no correlation between hereditary 25OHD levels and BMD, procollagen type 1 N-terminal propeptide (P1NP), carboxy-terminal telopeptide of type 1 collagen (CTX), and vertebral fractures (all *P* > 0.05) in all participants. In participants aged 55–92 years, MR analysis revealed that low serum 25OHD levels (odds ratio [OR] = 1.38; 95% confidence interval [CI]: 1.07–1.79; *P* = 0.014) are causally associated with increased P1NP levels. In sensitivity analysis, MR-Egger regression and the Weighted-median estimator indicated no significant pleiotropy.

**Conclusions:**

Our findings suggest that baseline 25OHD levels are unlikely to have a large effect on risk of vertebral fractures. Larger MR studies and randomized controlled trials are required to investigate small effects.

## Introduction

Vitamin D deficiency has emerged as a global public health concern.^[1]^ Serum 25-hydroxyvitamin D (25OHD) levels reflect the body’s vitamin D stores; it is the main clinical indicator of vitamin D status.^[2]^ Vitamin D deficiency can impair the absorption of calcium and phosphorus, both of which are critical for bone health, and can therefore lead to low bone mass and osteoporotic fractures; severe deficiency can lead to defective bone mineralization, resulting in rickets and osteochondrosis.^[3]^ A study conducted at our center reported that the mean serum 25OHD level of 4822 postmenopausal women was low at 24.2 μg/L was had a significant positive correlation with total hip bone mineral density (BMD).^[4]^ Together, these findings point to an association between vitamin D and osteoporosis.

The relationship between vitamin D supplementation and bone health remains controversial. Some studies have confirmed that vitamin D supplementation can prevent osteoporotic fractures, reduce bone loss in the thoracolumbar spine, and lower the risk of hip fractures,^[5,6]^ whereas others have found no direct causal effect on improving BMD and preventing osteoporotic fractures.^[7,8]^ These discrepancies may arise due to various factors, such as differences in supplementation dose, duration, and frequency,^[9]^ as well as the diversity of study areas, populations, and methods. Whether a significant correlation exists between vitamin D levels, BMD, and osteoporotic fractures remains inconclusive.

Mendelian randomization (MR) is an instrumental variable (IV) method that uses genetic variants, specifically single-nucleotide polymorphisms (SNPs), as instruments to infer the causal relationship between two traits, with the advantage of minimizing confounding and reverse causality.^[10, 11, 12, 13]^ Our center previously published an MR study of 1824 postmenopausal women in 2015,^[14]^ which included 10 SNPs associated with serum 25OHD levels, but did not observe a causal association between genetically low serum 25OHD and BMD and bone turnover markers (BTMs); no osteoporotic fracture population was included in that study. In the current study, we expand on this work by including 15 SNPs in 2261 participants and examining the possible causal associations between serum 25OHD levels and BMD, BTMs, and vertebral fractures by using MR. The findings are expected to provide early screening and identification of risk groups, clarify the risk factors for vitamin D deficiency and osteoporosis and bone health, identify the utility of vitamin D supplementation individuals at risk of osteoporosis and fracture, and guide clinical diagnosis and treatment and policy formulation.

## Materials and methods

### Study participants

We recruited 2061 participants aged 20–92 years from the healthy community population of Xiangshan Community, Zhejiang Province (community participants), and 200 patients with osteoporosis attending the outpatient clinic at our department of osteoporosis from a local population in Shanghai. All participants were permanent residents of the middle east coast of China.

The inclusion criteria were as follows: (1) no history of diseases affecting bone metabolism or vitamin D metabolism (except osteoporosis) or history of medication use; (2) complete biochemical data with no serious liver or kidney function abnormalities and no malignant tumors; (3) natural menopause.

For patients with fracture, the circumstances surrounding each fracture were recorded through personal interview. X-rays were taken of patients attending the department. We included only patients with low-trauma fractures caused by falls from standing height or less. Vertebral fractures were limited to those presenting to clinical attention. We excluded participants with an underlying disorder, such as cancer or bone disease, that could cause a pathological fracture.

The study was approved by the ethics committee of Sixth Hospital affiliated with Shanghai Jiaotong University School of Medicine (Approval number: 2020-178). The study aims and procedures were explained in detail to all participants, and written inform consent was obtained from all participants before enrollment.

### BMD measurements

BMD of lumbar vertebrae L1–L4, left femoral neck, and total hip was measured in community participants by using a dual-energy X-ray absorptiometry scanner (Discovery Wi, Hologic, USA). Right hip BMD was measured only in patients with a history of left hip fracture or surgery. The bone densitometer used was calibrated daily during the study, and all BMD measurements were performed by the same skilled technician. Body weight and height were measured using a calibrated balance beam scale and a calibrated stadiometer, and body mass index (BMI) was calculated as weight divided by height squared (kg/m^2^).

### X-rays

To assess the presence of vertebral fractures, X-rays of the thoracolumbar spine in the anteroposterior and lateral views and of the pelvis in anteroposterior view were obtained from all patients attending the department.

### Selection of IVs for MR analyses

We conducted a one-sample MR study to investigate causal relationships between serum 25OHD levels and BMD, BTMs, and vertebral fractures.

Genetic variants (SNPs) associated with 25OHD were selected as IVs based on genome-wide significance (*P* < 5×10^−8^) from prior genome-wide association studies (GWAS) and previous association studies in Chinese populations.^[15, 16, 17, 18, 19, 20]^ A total of 15 SNPs within the vitamin D metabolic pathway (*i.e*., *GC*-rs4588, *GC*-rs7041, *GC*-rs2282679, *GC*-rs1155563, *NADSYN1*-rs2276360, *NADSYN1*-rs12785878, *CYP2R1*-rs2060793, *CYP2R1*-rs10741657, *CYP2R1*-rs10766197 and *CYP24A1*-rs6013897) were selected as potential IVs for the MR analyses.

Blood samples were collected from all participants, and genomic deoxyribonucleic acid (DNA) was extracted and purified from peripheral blood leukocytes by using a QuickGene DNA whole blood kit L by Nucleic Acid Isolation System (QuickGene-610 L, FUJI FILM, Japan). Genotyping was performed with an ABI PRISM SNaPshot multiplex kit (Applied Biosystems), an Mx3000p real-time PCR system (Stratagene), and GeneMapper 4.0 (Applied Biosystems). Genotype frequencies were estimated on the basis of the Hardy-Weinberg Equilibrium (HWE) with a chi-square test to detect genotyping errors.

All candidate SNPs underwent rigorous quality control using PLINK (version 1.9) with the following criteria: (1) minor allele frequency (MAF) filtering. SNPs with MAF < 1% were excluded; (2) HWE testing. Variants with HWE *P* value < 1×10^−6^ were removed; (3) linkage disequilibrium pruning. SNPs were clumped at *R*^2^ < 0.01 within 10,000 kb windows using the 1000 Genomes Project as reference.

### Statistical analysis

We assessed the distribution of all continuous variables and excluded the extreme values (> 3.5 standard deviations [SD] from the mean; affecting < 1% of all data points). Normally distributed variables are reported as the means ± SD, and nonnormally distributed variables are reported as medians (25th–75th percentiles or interquartile range [IQR]). Natural logarithmic transformation was used for the skewed variables to approximate normality for the subsequent data analyses. Missing values were handled using the multiple imputation method.

We performed comprehensive association analyses in the full study population to evaluate (1) exposure associations, defined as the relationship between each SNP and circulating 25OHD levels, and (2) outcome associations, defined as SNP associations with BMD, BTMs, and vertebral fracture. Only SNPs meeting strict criteria of genome-wide significance (*P* < 5×10^−8^) and demonstrating sufficient instrument strength (*F* statistic > 10) were retained for final MR analyses. All analyses were adjusted for age, sex and BMI.

For each variant included in the genetic instrument, the proportion of variance explained *R^2^* was computed using the ‘get_r_from_pn’ function in the TwoSampleMR package. *F* statistics were subsequently determined for each variant, using the formula where *N* is the sample size of the exposure GWAS and k corresponds to the number of IVs. This was used to the efficacy of the chosen instruments.


$$F-statistics=\frac{N-k-1}{k}\times \frac{R^{2}}{1-R^{2}}$$

**Figure1:**
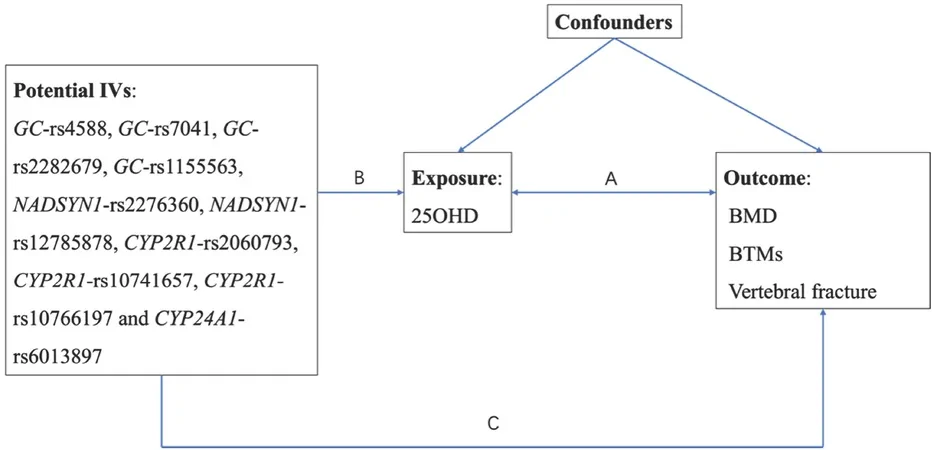
Overview of this MR study design. A indicates the cross-sectional association between exposure factors and outcome variables. B indicates the effect of genetic variation on exposure factors. C indicates the effect of the level of exposure factor determined using genetic variation on the outcome variable. MR, Mendelian randomization; BMD, bone mineral density; BTMs, bone turnover markers; 25OHD, 25-hydroxyvitamin D; IV, instrumental variable.

The primary MR analysis was conducted using a one-sample framework with the following specifications: (1) Exposure. Serum 25OHD concentration; (2) Outcomes. BMD, BTMs, and vertebral fracture; (3) IVs. Quality-controlled SNPs from vitamin D pathway genes. The framework is described in Figure 1.

MR estimates for the associations of genetically predicted 25OHD concentrations with BMD, BTMs, and vertebral fracture were obtained using the conventional inverse-variance weighted method and complemented with MR-Egger methods.

Data for external validation were sourced from publicly available databases. BMD GWAS data from the GWAS Catalog (https://www.ebi.ac.uk/gwas/publications/26367794) for femoral neck and lumbar spine, vitamin D GWAS data from OpenGWAS (ID: ukb-e-100021_AFR), and spine fracture data from OpenGWAS (ID: ukb-b-873).

All statistical analyses were performed using R (version 4.3.0), with MR and sensitivity analyses conducted using the TwoSampleMR package.^[21]^

## Results

### Basic characteristics of the study population

We enrolled 2261 participants, and their clinicodemographic and biochemical characteristics are presented in Table 1. Their median (IQR) age was 63 (50–68) years, median BMI was 22.9 (21.0–25.0) kg/m^2^, and median serum 25OHD level was 29 (23–37) μg/L. A total of 328 participants had a history of vertebral fracture (74 community participants and 254 outpatients). In this study, BMD analyses were restricted to the Xiangshan Community population because the outpatient cohort was assessed using a different bone densitometer.

**Table 1:** Basic characteristics of all the study participants

Item	Male (*n* = 743)	Female (*n* = 1518)	All participants (*n* = 2261)	Minimum values	Maximum values
Age (years)	64 (53–69)	62 (49–68)	63 (50–68)	20	92
Height (cm)	170 (165–173)	158 (155–162)	160 (157–167)	125	188
Weight (kg)	67.0 (60.0–74.0)	56.0 (51.5–63.0)	60.0 (53.7–67.0)	32.4	100
BMI (kg/m^2^)	23.4 (21.3–25.4)	22.7 (20.8–25.0)	22.9 (21.0–25.0)	15.1	32.0
Ca (mmol/L)	2.39 (2.33–2.45)	2.40 (2.35–2.45)	2.40 (2.34–2.45)	2.14	2.62
P (mmol/L)	1.12 (1.02–1.24)	1.26 (1.16–1.37)	1.23 (1.11–1.34)	0.80	1.64
P1NP (μg/L)	45.6 (36.1–58.5)	54.0 (40.3–69.9)	50.7 (38.8–66.3)	18.1	133.5
CTX (μg/L)	120 (70–170)	130 (70–220)	130 (70–200)	30	1005
ALB (g/L)	47.9 (45.4–50.4)	47.2 (45.0–49.3)	47.3 (45.1–49.7)	36.3	54.9
Cr (μmol/L)	78 (71–86)	61 (55–68)	66 (58–76)	44	119
UA (μmol/L)	377 (319–429)	288 (249–335)	310 (262–377)	163	591
25OHD (μg/L)	36 (29–45)	27 (22–33)	29 (23–37)	9	64
ALP (U/L)	69 (59–82)	70 (56–87)	70 (57–85)	24	170
L1–L4 BMD (g/cm^2^)	0.978 (0.890–1.100)	0.868 (0.746–0.992)	0.923 (0.802–1.038)	0.414	2.087
Femoral neck BMD (g/cm^2^)	0.783 (0.703–0.867)	0.679 (0.596–0.772)	0.722 (0.628–0.817)	0.363	1.575
Total hip BMD (g/cm^2^)	0.936 (0.851–1.023)	0.810 (0.717–0.903)	0.851 (0.752–0.954)	0.413	1.365
Vertebral fracture	59	259	328	-	-

Data were presented as medians (25th–75th percentiles). BMD, bone mineral density; BMI, body mass index; Ca, serum total calcium; P, serum phosphate; P1NP, procollagen type 1 N-terminal propeptide; CTX, carboxy-terminal telopeptide of type 1 collagen; ALB, albumin; Cr: serum creatinine; UA, uric acid; 25OHD, 25-hydroxyvitamin D; ALP, serum alkaline phosphatase; L1–L4 BMD, *n* = 2015; femoral neck BMD, *n* = 2012; total hip BMD, *n* = 2014.

Among participants aged 55–92 years (*n* = 1580; 1030 postmenopausal women and 550 men), the median (IQR) age was 66 (63–70), and median serum 25OHD level was 31 (25–39) μg/L. Moreover, 328 participants had a history of vertebral fracture (Table 2).

**Table 2:** Basic characteristics of study participants aged 55–92 years

Item	Participants (*n* = 1580)	Minimum values	Maximum values
Age (years)	66 (63–70)	55	92
Height (cm)	160.0 (156.0–166.0)	134.3	185.o
Weight (kg)	60.0 (55.0–67.0)	32.4	95
BMI (kg/m^2^)	23.4 (21.5–25.4)	15.1	32.0
Ca (mmol/L)	2.40 (2.34–2.45)	2.14	2.62
P (mmol/L)	1.19 (1.07–1.30)	0.80	1.64
P1NP (μg/L)	53.2 (40.0–68.7)	18.1	133.5
CTX (μg/L)	160 (110–230)	30	1005
ALB (g/L)	46.9 (44.4–49.4)	36.3	54.9
Cr (μmol/L)	66 (58–77)	44	119
UA (μmol/L)	316 (267–378)	163	591
25OHD (μg/L)	31 (25–39)	9	64
ALP (U/L)	75 (63–90)	26	170
L1–L4 BMD (g/cm^2^)	0.871 (0.754–0.993)	0.414	1.616
Femoral neck BMD (g/cm^2^)	0.685 (0.600–0.777)	0.363	1.559
Total hip BMD (g/cm^2^)	0.835 ± 0.144	0.413	1.365

Data were presented as medians (25th–75th percentiles). BMD, bone mineral density; BMI, body mass index; Ca, serum total calcium; P, serum phosphate; P1NP, procollagen type 1 N-terminal propeptide; CTX, carboxy-terminal telopeptide of type 1 collagen; ALB, albumin; Cr: serum creatinine; UA, uric acid; 25OHD, 25-hydroxyvitamin D; ALP, serum alkaline phosphatase; L1–L4 BMD, *n* = 1343; femoral neck BMD, *n* = 1341; total hip BMD, *n* = 1342.

### Associations between SNPs and clinical traits

The basic information of the selected SNP loci is presented in Table 3. Their MAFs were consistent with the HapMap-CHB reference database, and the genotyping rate of all the loci was > 90%; all SNPs conformed to the Hardy–Weinberg law of equilibrium.

**Table 3:** Basic information on SNP associated with 25OHD metabolism

CHR	Gene	SNP	A1	A2	MAF	NCHROBS
4	GC	rs1155563	C	T	0.4262	4514
4	GC	rs2282679	G	T	0.3355	4522
4	GC	rs4588	T	G	0.3432	4508
4	GC	rs7041	C	A	0.2610	4506
11	CYP2R1	rs10741657	A	G	0.3529	4522
11	CYP2R1	rs10766197	A	G	0.3605	4522
11	NADSYN1	rs12785878	T	G	0.4558	4522
11	CYP2R1	rs12794714	A	G	0.3673	4520
11	CYP2R1	rs2060793	A	G	0.3534	4522
11	NADSYN1	rs2276360	C	G	0.4553	4522
12	AMDHD1	rs10745742	C	T	0.4766	4522
14	SEC23A	rs8018720	G	C	0.3699	4520
20	CYP24A1	rs17216707	C	T	0.0394	4522
20	SSTR4	rs2207173	G	A	0.4241	4522
20	CYP24A1	rs6013897	A	T	0.1515	4522

25OHD, 25-hydroxyvitamin D; CHR, chromosome; SNP, single-nucleotide polymorphism; A1, allele 1 code, minor allele; A2, allele 2 code, major allele; MAF, minor allele frequency; NCHROBS, Nonmissing allele count.

As a prerequisite for MR analysis, we examined the association between these SNPs and clinical variables (25OHD, BMD, P1NP, CTX, and vertebral fracture), adjusted for age, sex, and BMI. In the overall cohort, the association analysis between SNP and 25OHD indicated that rs2282679, rs4588, rs7041, rs10766197, and rs12794714 meet the genome-wide significance (*P* < 5×10^−8^; Table 4) and had no statistical correlation with BMD, P1NP, CTX, and vertebral fracture (Table 5). Therefore, these five SNPs were used as the IVs for the subsequent MR analyses in the overall cohort. Among participants aged 55–92 years, rs2282679, rs4588, rs10766197, and rs12794714 were included as the IVs (Tables 6 and 7).

**Table 4:** Effect of SNP genotype on 25OHD levels in all participants (*n* = 2261)

SNP	Beta	*P*	SE
rs1155563	−1.409	<0.001	0.267
rs2282679	−2.434	<5×10^−8^	0.276
rs4588	−2.673	<5×10^−8^	0.273
rs7041	1.791	<5×10^−8^	0.300
rs10741657	1.083	0.002	0.282
rs10766197	−2.159	<5×10^−8^	0.278
rs12785878	0.932	0.008	0.268
rs12794714	−2.188	<5×10^−8^	0.278
rs2060793	1.066	0.002	0.282
rs2276360	0.961	0.005	0.268
rs10745742	−0.229	1.000	0.267
rs8018720	0.708	0.182	0.282
rs17216707	0.090	1.000	0.695
rs2207173	−0.287	1.000	0.267
rs6013897	0.070	1.000	0.369

25OHD, 25-hydroxyvitamin D; SNP, single-nucleotide polymorphism; Beta, regression coefficient; SE, standard error. Bonferroni method was used to control the family-wise error rate when multiple hypotheses tests were performed. The analyses were performed under additive models by adjusting for age, sex, and body mass index (BMI).

**Table 5:** Correlation of SNP loci with clinical variables in all participants (*n* = 2261)

Item	rs2282679		rs4588		rs7041		rs10766197		rs12794714	
	Beta	*P*	Beta	*P*	Beta	*P*	Beta	*P*	Beta	*P*
L1–L4 BMD	–0.001	1	–0.002	1	–0.002	1	–0.003	1	–0.005	1
Femoral neck BMD	0.001	1	<0.001	1	–0.002	1	–0.001	1	–0.003	1
Total hip BMD	<0.001	1	<–0.001	1	–0.002	1	–0.001	1	–0.003	1
P1NP	–0.690	1	–0.529	1	0.498	1	–0.411	1	–0.504	1
CTX	–4.750	1	4.339	1	–1.544	1	–2.601	1	–5.037	1
Vertebral fracture	–0.100	1	0.013	1	0.008	1	0.025	1	0.013	1

BMD, bone mineral density; P1NP, procollagen type 1 N-terminal propeptide; CTX, carboxy-terminal telopeptide of type 1 collagen; SNP, single-nucleotide polymorphism. L1–L4 BMD, *n* = 2015; femoral neck BMD, *n* = 2012; total hip BMD, *n* = 2014.

**Table 6:** Effect of SNP genotype on 25OHD levels in participants aged 55-92 years (*n* = 1580)

CHR	SNP	Beta	*P*	SE
4	rs1155563	–1.650	<0.001	0.337
4	rs2282679	–2.703	<5×10^–8^	0.350
4	rs4588	–2.962	<5×10^–8^	0.345
4	rs7041	1.792	<0.001	0.374
11	rs10741657	1.682	<0.001	0.356
11	rs10766197	–2.527	<5×10^–8^	0.351
11	rs12785878	1.085	0.021	0.339
11	rs12794714	–2.625	< 5×10^–8^	0.350
11	rs2060793	1.663	<0.001	0.355
11	rs2276360	1.117	0.015	0.339
12	rs10745742	–0.159	1.000	0.337
14	rs8018720	0.769	0.514	0.363
20	rs17216707	–0.008	1.000	0.867
20	rs2207173	–0.318	1.000	0.332
20	rs6013897	0.435	1.000	0.465

25OHD, 25-hydroxyvitamin D; SNP, single-nucleotide polymorphism; Beta, regression coefficient; SE, standard error; CHR, chromosome. Bonferroni method was used to control the family-wise error rate when multiple hypotheses tests were performed. The analyses were performed under additive models by adjusting for age, sex, and body mass index (BMI).

**Table 7:** Correlation of SNP loci with clinical variables in participants aged 55-92 years (*n* = 1580)

Item	rs2282679		rs4588		rs10766197		rs12794714	
	Beta	*P*	Beta	*P*	Beta	*P*	Beta	*P*
L1–L4 BMD	–0.005	1	–0.004	1	–0.003	1	–0.005	1
Femoral neck BMD	0.001	1	0.001	1	–0.003	1	–0.005	1
Total hip BMD	–0.002	1	–0.002	1	–0.003	1	–0.005	1
P1NP	–1.060	1	–0.904	1	–0.721	1	–0.828	1
CTX	–5.737	1	7.008	1	–3.941	1	–7.106	1
Vertebral fracture	–0.106	1	0.016	1	–0.020	1	–0.028	1

BMD, bone mineral density; P1NP, procollagen type 1 N-terminal propeptide; CTX, carboxy-terminal telopeptide of type 1 collagen; Beta, regression coefficient. L1–L4 BMD, *n* = 1343; femoral neck BMD, *n* = 1341; total hip BMD, *n* = 1342.

### Evaluation of causal associations between 25OHD levels and clinical outcomes

The weak IV test was first performed for each SNP, and the results are presented in Table 8 and Table 9. All SNPs were included in the MR analysis.

**Table 8:** Weak IV test in all participants

SNP	Beta	*P*	SE	*R* ^2^	*F* statistic
rs2282679	–2.434	<0.001	0.276	0.176	96.27
rs4588	–2.673	<0.001	0.273	0.196	110.07
rs7041	1.791	<0.001	0.300	0.115	58.75
rs10766197	–2.159	<0.001	0.278	0.154	81.96
rs12794714	–2.188	<0.001	0.278	0.156	83.43

*P*, Bonferroni-corrected *P* value; IV, instrumental variable; SNP, single-nucleotide polymorphism; SE, standard error.

**Table 9:** Weak IV test in participants aged 55-92 years

SNP	Beta	*P*	SE	*R* ^2^	*F* statistic
rs2282679	–2.703	<0.001	0.350	0.182	87.71
rs4588	–2.962	<0.001	0.345	0.204	100.73
rs10766197	–2.527	<0.001	0.351	0.169	80.01
rs12794714	–2.625	<0.001	0.350	0.177	84.46

*P*, Bonferroni-corrected *P*-value; IV, instrumental variable; SNP, single-nucleotide polymorphism; SE, standard error.

In the overall cohort, MR analysis indicated no correlation between hereditary 25OHD levels and BMD, serum P1NP, CTX, and vertebral fractures (all *P* > 0.05) (Table 10 and Supplementary Figure S1). In participants aged 55–92 years, low serum 25OHD levels (odds ratio [OR] = 1.38; confidence interval [CI]: 1.07-1.79; *P* = 0.014) were causally associated with increased P1NP levels (Table 10 and Supplementary Figure S2). Sensitivity analysis using MR-Egger regression and the weighted-median estimator indicated no significant pleiotropy. The causal relationships between vitamin D and BMD and vertebral fractures were validated using publicly available databases, with results presented in Supplementary Table S1.

**Table 10:** MR analysis effect estimates for the association between serum 25OHD levels and BMD, BTMs, vertebral fractures

Exposure	Outcome	Method	Number of SNPs	OR	95%CI	*P*
25OHD (entire cohort)	P1NP	IVW	5	1.26	0.99–1.60	0.059
	CTX			1.73	0.26–11.42	0.568
	L1–L4 BMD			1.00	0.99–1.00	0.335
	Femoral neck BMD			1.00	0.99–1.00	0.934
	Total hip BMD			1.00	0.99–1.00	0.665
	Vertebral fracture			1.00	0.97–1.04	0.798
25OHD (age 55–92	P1NP	IVW	4	1.38	1.07–1.79	0.014
years)	CTX			2.17	0.19–24.98	0.533
	L1–L4 BMD			1.00	0.99–1.00	0.090
	Femoral neck BMD			1.00	0.99–1.00	0.486
	Total hip BMD			1.00	0.99–1.00	0.134
	Vertebral fracture			1.00	0.97–1.04	0.820

25OHD, 25-hydroxyvitamin D; BMD, bone mineral density; BTMs, bone turnover markers; P1NP, procollagen type 1 N-terminal propeptide; CTX, carboxy-terminal telopeptide of type 1 collagen; IVW, inverse-variance weighted; OR, odds ratio; CI, confidence interval. Entire cohort: L1–L4 BMD, *n* = 2015; femoral neck BMD, *n* = 2012; total hip BMD, *n* = 2014. Age 55–92 years: L1–L4 BMD, *n* = 1343; femoral neck BMD, *n* = 1341; total hip BMD, *n* = 1342.

## Discussion

In this MR study, we examined the causal relationships between serum 25OHD and BMD (restricted to the Xiangshan Community population), BTMs, and vertebral fractures was performed in 2261 participants aged 20–92 years, of whom 1580 were aged 55–92 years. MR analysis indicated that genetically low serum 25OHD levels were causally associated with higher serum P1NP levels in participants aged 55–92 years. No causal relationship was observed between hereditary low serum 25OHD levels and the occurrence of vertebral fractures.

Serum 25OHD levels are influenced by diet or supplementation intake, but genetic factors also play a vital role. Mutations in genes in the metabolic pathway of 25OHD can affect 25OHD levels *in vivo*. In recent years, a number of GWAS of serum 25OHD have been conducted in populations of European ancestry, the largest of which included 79, 366 individuals.^[20,22,23]^ These studies identified six common genetic variants associated with 25OHD levels. These SNPs were located at or near genes related to vitamin D metabolism, including vitamin D synthesis (rs12785878 locus of the *DHCR7*/*NADSYN* gene encoding 7-dehydrocholesterol reductase and *CYP2R1* gene encoding 25-hydroxylase rs10741657 site), transport (rs2282679 site of the *GC* gene encoding a vitamin D binding protein) and degradation (rs17216707 site of the *CYP24A1* gene encoding a 24-hydroxylase) processes, as well as SNPs outside of known vitamin D metabolic pathways, such as rs8018720 on the *SEC23A* gene (Sec23 homolog A, a component of shell protein complex II) and rs10745742 on the *AMDHD1* gene (amidase-containing domain 1, an enzyme involved in the histidine, lysine, phenylalanine, tyrosine, proline, and tryptophan catabolic pathways). Other SNPs associated with 25OHD identified by GWAS studies are rs117913124) ^[24]^ and rs 12794714 ^[18]^ for the *CYP2R1* gene and rs2207173^[19]^ for *FOXA2*/*SSTR4*. Our previous studies on vitamin D-related genetics in Chinese populations also provide an important basis for further analysis of the effect of vitamin D status on bone health in follow-up. We genotyped 96 SNPs in 15 genes along the vitamin D metabolic pathway in 2897 people with Han Chinese ethnicity in Shanghai and noted that two SNP loci in the *GC* gene (rs2282679 and rs4588) and one SNP locus in the *CYP2R1* gene (rs10766197) were strongly associated with serum 25OHD levels.^[16]^ Lu *et al*.^[17]^ also observed that several common SNP loci on both the GC gene and the *DHCR7*/*NADSYN1* gene were significantly associated with 25OHD levels in Han Chinese populations in Beijing and Shanghai. Taken together, candidate gene variants clearly affect 25OHD levels, providing a basis for our MR analysis.

In the present study, genetically predicted lower serum 25OHD levels were associated with elevated serum P1NP levels in adults aged 55–92 years, suggesting a potential causal relationship between vitamin D deficiency and increased bone turnover in older adults. Deficiency-induced hypocalcemia triggers secondary hyperparathyroidism, elevating parathyroid hormone (PTH) levels, thereby accelerating coupled bone turnover and increasing both resorption and formation markers.^[25]^ Concurrently, insufficient levels of active 1,25(OH)_₂_D impair bone matrix mineralization,^[26]^ the resulting accumulation of unmineralized osteoid may provoke a compensatory increase in osteoblast activity and collagen synthesis, reflected by higher P1NP.^[27]^ Furthermore, direct vitamin D receptor–mediated signaling in osteoblasts and osteocytes is disrupted in deficiency. This leads to osteocyte apoptosis^[26]^ and dysregulation of genes controlling osteoblast function and local remodeling signals, including the RANKL/ OPG axis, contributing to an anabolic bone state.^[28]^ Thus, elevated P1NP likely represents a composite of hormonal, mineral, and cellular disturbances stemming from vitamin D deficiency. The findings align with studies showing that vitamin D deficiency is associated with higher BTMs in elderly populations.^[29,30]^ The correlation between 25OHD and BTMs was reported mainly in vitamin-deficient groups^[29, 30, 31, 32]^ and P1NP levels were only suppressed by vitamin D treatment when baseline 25OHD was < 30 nmol/L in an elderly population.^[33]^ Taken together, these findings suggest that maintaining adequate vitamin D levels may help regulate bone metabolism in older adults.

MR analysis did not reveal a significant causal relationship between genetically predicted low serum 25OHD levels and vertebral fracture risk. Previous randomized controlled trials investigating the impact of vitamin D supplementation on vertebral fracture outcomes have yielded conflicting results. Among generally healthy European older adults (aged ≥70 years), vitamin D3 supplementation did not reduce vertebral fracture incidence.^[34]^ A U.K. trial found only a marginal reduction in spine fracture risk with 100,000 IU of vitamin D_3_ every 4 months compared with a placebo for 5 years.^[35]^ The Vitamin D and Omega-3 Trial (VITAL) trial tested the dose of 2000 IU vitamin D3 over a median follow-up of 5.3 years, included 25,871 participants (mean age 67 years) and reported no benefits for total, nonvertebral, or hip fracture risk.^[36]^ Vitamin D may primarily affect fracture risk only when deficiency is severe, whereas moderate insufficiency might not significantly impact vertebral fracture occurrence. This was observed in a prospective risk assessment study of osteoporosis conducted in Switzerland, where older women with 25OHD levels <20 μg/L were found to have an increased risk of fracture.^[37]^ In addition, isolated vitamin D supplementation, without addressing other risk factors (*e.g*., falls, muscle weakness, or calcium intake), may not substantially reduce vertebral fractures in older adults. A meta-analysis by Papadimitropoulos *et al*.^[38]^ concluded that vitamin D supplementation resulted in a significant 37% reduction in vertebral fracture risk; however, this analysis pooled studies comparing vitamin D + calcium with placebo, vitamin D alone with placebo, and vitamin D with calcium. Another meta-analysis including 68,500 patients^[7]^ reported that vitamin D supplementation alone did not prevent vertebral and hip fractures, regardless of gender and history of fracture, whereas concomitant supplementation with vitamin D and calcium reduced the risk of hip and vertebral fractures.

Current guidelines also do not provide a single, universally accepted 25OHD threshold for clinical intervention. The 2024 American Endocrine Society clinical practice guideline no longer recommends using specific 25OHD levels to define vitamin D sufficiency, insufficiency, or deficiency and, instead, recommends that vitamin D supplementation be considered based on individual circumstances and potential health benefits. For adults aged ≥75 years, empirical supplementation is recommended to reduce overall mortality and decrease the risk of osteoporosis and fractures, particularly in those with serum 25OHD levels < 20 μg/L.^[39]^ However, maintaining sufficient 25OHD levels is vital for overall musculoskeletal health.

A strength of our study is that the MR analysis effectively mitigates reverse causality and confounding factors.

Nevertheless, this study has some limitations. First, PTH testing was not included in the analysis because PTH levels may have been affected due to the long-distance transportation of blood samples to Shanghai. Second, 25OHD levels are also affected by the season of blood collection, which was not considered in this study. Third, the vertebral fracture cohort were mainly recruited from outpatient settings, which may introduce selection bias. Although our MR analyses conducted separately in outpatient and community populations yielded consistent conclusions, given the small sample size of outpatient patients, future replication in a more representative population-based cohort is warranted to confirm our findings.

In conclusion, our findings indicated that genetically low serum 25OHD levels were causally associated with higher serum P1NP levels in the MR analysis. No causal evidence was found for an association between low serum 25OHD levels and the occurrence of vertebral fractures. Further investigation in larger, population-based MR studies and randomized controlled trials is needed to determine the exact risk correlation between 25OHD, BMD, and fractures.

## Supplementary Material

Supplementary Material Details
