# Supplementary material for: Circulating serum 25-hydroxyvitamin D levels and bone mineral density and osteoporotic vertebral fractures: A Mendelian randomization study
Source: J Transl Int Med. 2026 Jun 22;14(4):610–9. doi: 10.1515/jtim-2026-0058 (PMC13509146; doi:10.1515/jtim-2026-0058)
Supplement: Supplementary file 1 [file jtim-2026-0058_sm.pdf]

Supplementary materials

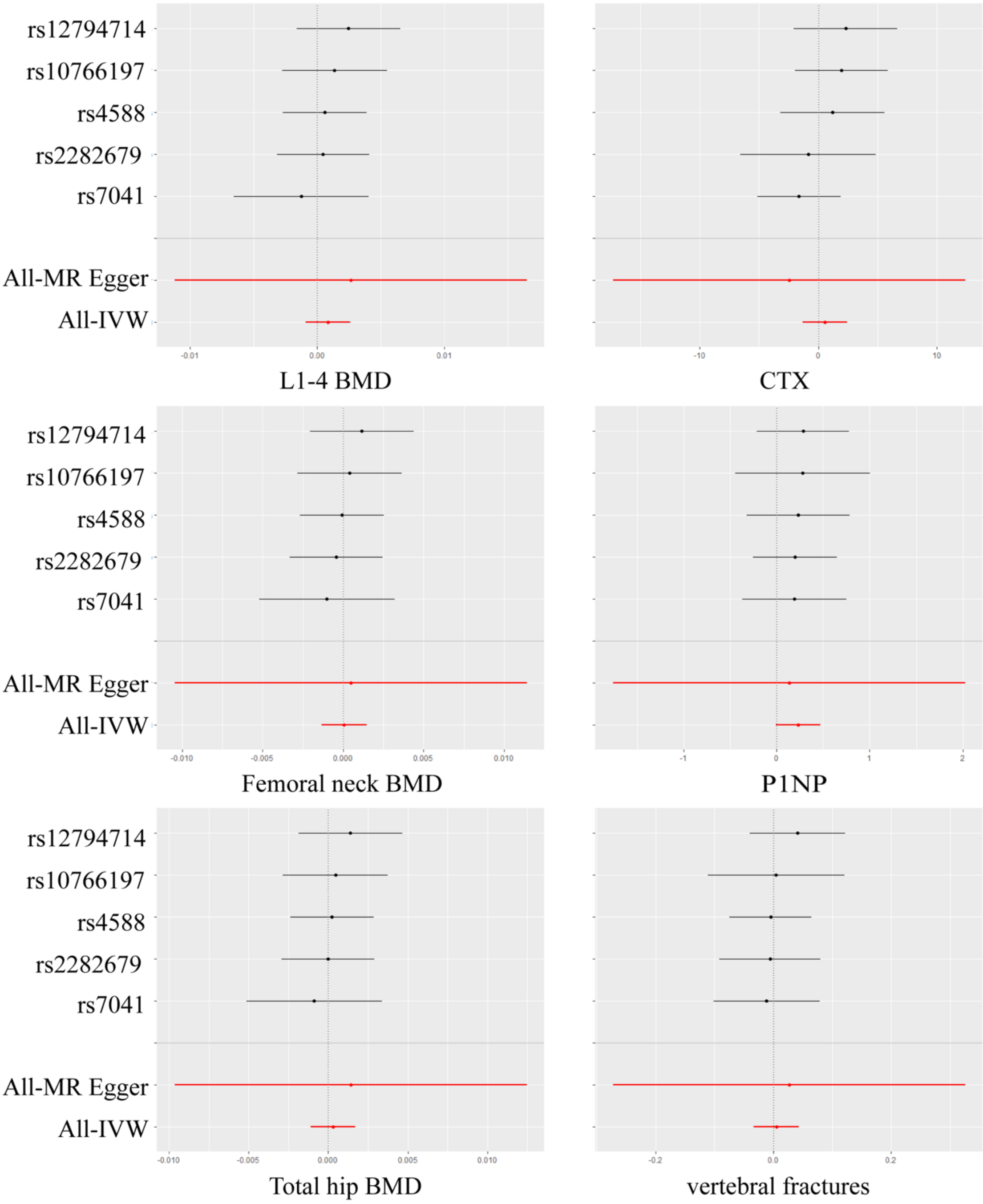

Supplementary Figure S1: Forest plot for causal effects of 25OHD on BMD, serum P1NP, CTX, and vertebral fractures in all subjects. BMD, bone mineral density; 25OHD, 25-hydroxyvitamin D; P1NP, procollagen type 1 N-terminal propeptide; CTX, carboxy-terminal telopeptide of type 1 collagen; MR, Mendelian randomization; IVW, inverse variance weighted.

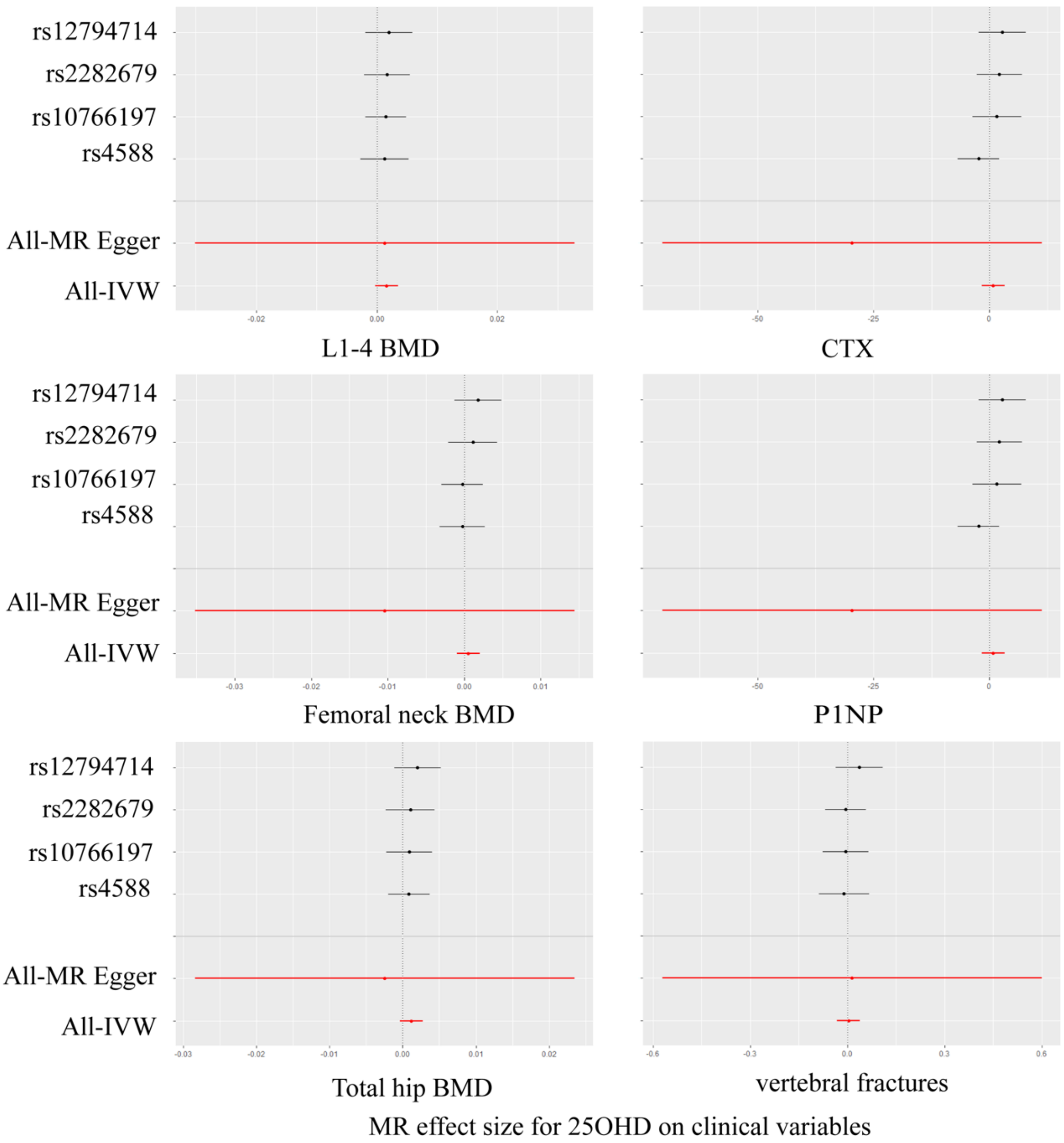

Supplementary Figure S2: Forest plot for causal effects of 25OHD on BMD, serum P1NP, CTX, and vertebral fractures in participants aged 55-92 years. BMD, bone mineral density; 25OHD, 25-hydroxyvitamin D; P1NP, procollagen type 1 N-terminal propeptide; CTX, carboxy-terminal telopeptide of type 1 collagen; MR, Mendelian randomization; IVW, inverse variance weighted.

Supplementary Table S1: MR analysis effect estimates for the association between serum 25OHD levels and BMD, spine fractures

**Outcome**	**Exposure**	**Method**	**Number of SNPs**	**OR**	**95%CI**	***P***

Spine Fracture	Vitamin D	IVW	6	1.000	1.999-1.000	0.057
lumber spine BMD	Vitamin D	IVW	22	1.005	1.001-1.009	0.028
Femoral neck BMD	Vitamin D	IVW	23	1.003	0.991-1.015	0.624

BMD, bone mineral density; 25OHD, 25-hydroxyvitamin D; MR, Mendelian randomization; IVW, inverse variance weighted; SNPs, single-nucleotide polymorphisms; OR, odds ratio; CI, confidence interval.
